# Exploring the Incidence and Risk Factors of Dyslipidemia in Patients with Severe Acne Vulgaris on Systemic Isotretinoin Therapy: Findings from a Prospective Study

**DOI:** 10.3390/medicina61030439

**Published:** 2025-02-28

**Authors:** Jihan Muhaidat, Leen Alhuneafat, Rand Asfar, Firas Al-Qarqaz, Diala Alshiyab, Laith Alhuneafat

**Affiliations:** 1 Department of Dermatology, Jordan University of Science and Technology, P.O.Box 3030, Irbid 22110, Jordan; leen.alhuneafat@gmail.com (L.A.); randasfar@gmail.com (R.A.); fqarqaz@just.edu.jo (F.A.-Q.); dmalshiyab@just.edu.jo (D.A.); 2 Cardiovascular Division, University of Minnesota, Minneapolis, MN 55455, USA; alhun005@umn.edu

**Keywords:** dyslipidemia, HLD, DLP, hyperlipidemia, isotretinoin, acne vulgaris

## Abstract

*Background and Objectives*: Oral isotretinoin has revolutionized the treatment of severe acne vulgaris. Isotretinoin is associated with multiple adverse effects, one of which is dyslipidemia (DLP). *Materials and Methods*: This single-center prospective study recruited 498 patients who were eligible for isotretinoin for severe acne. Risk factors for hyperlipidemia and serum lipids were assessed at baseline. Patients received daily doses ranging from 0.25 to 1 mg/kg of their body weight, and their fasting serum lipids were checked regularly until they reached a cumulative dose of 120–150 mg/kg. Our primary objective is to investigate dyslipidemia incidence and predictors, while the secondary objective is to assess the impact of dose reduction on lipid panels. *Results*: Our sample was primarily female (n = 380, 76.3%), with a normal Body Mass Index (23.2 ± 4.0) and a mean age of 20.7 (±4.1) years. About 72.5% had a family history of acne, 17.1% a family history of dyslipidemia. Around 17.3% reported tobacco use. A total of 57 (11.4%) patients on isotretinoin developed DLP. Smoking was independently associated with a higher risk of dyslipidemia (OR 1.97, 95% CI [1.01, 3.82], *p* = 0.046). The mean onset of DLP was at 3.23 (±2.13) months. A total of 52 patients out of the 57 had a dose reduction of 10 mg (n = 5) or 20 mg (n = 47). A dose reduction of 50% was found to significantly improve triglyceride levels. *Conclusions*: More than 1 out of 10 patients on isotretinoin developed DLP. Tobacco use was significantly associated with developing DLP. Dose reduction significantly impacted a decrease in triglyceride levels.

## 1. Introduction

Isotretinoin, a synthetic vitamin A derivative, is a highly effective medication commonly used to treat severe acne vulgaris [1]. While isotretinoin has been shown to be highly effective in treating acne, it has also been associated with several adverse effects, including dyslipidemia, which is defined as an abnormal level of lipids in the blood [2]. Dyslipidemia is a major risk factor for the development of cardiovascular disease; therefore, patients undergo routine monitoring of lipids, as often as monthly [3].

Isotretinoin is thought to impact the liver’s homeostasis of lipids, causing dyslipidemia in a significant portion of patients being treated for acne [4]. Lipid abnormalities have primarily been related to an increase in mean serum triglyceride and cholesterol levels [1]. Dyslipidemia is not precluded by normal baseline serum lipid readings in patients receiving isotretinoin [5,6]. The increases are proportionate to the drug’s dose, but not to pre-treatment triglyceride or cholesterol levels [5]. Fortunately, the changes in lipid metabolism associated with isotretinoin are generally reversible, and most patients return to their baseline lipid levels after discontinuing the medication [7].

Even though lab monitoring practices have been the focus of multiple studies over the last two decades, there is still confusion about the type and frequency of lab monitoring ordered in ordinary clinical practice for patients taking isotretinoin. After the first few weeks of treatment, there is no clear evidence that increasing cumulative dosages of isotretinoin results in increased cholesterol levels [8].

There is limited information on the risk factors that may contribute to the development of dyslipidemia in individuals taking isotretinoin. Therefore, the aim of this study is to examine the rate of dyslipidemia in patients taking isotretinoin for acne vulgaris and to identify the risk factors associated with developing the condition. Currently, there is limited information on the effect of dose reduction on dyslipidemia in patients taking isotretinoin. Therefore, this study also aims to explore the impact of dose reduction in individuals who develop dyslipidemia.

## 2. Materials and Methods

### 2.1. Population

Patients presenting to a single-center academic center with acne vulgaris being considered for isotretinoin therapy were enrolled in the study from June 2022 to April 2023. Patients with baseline lipid abnormalities or individuals previously diagnosed with dyslipidemia were excluded from the study. Specifically, individuals with high-density lipoprotein (HDL) levels below 1.04 mmol/L for males and 1.29 mmol/L for females, low-density lipoprotein (LDL) levels exceeding 3.37 mmol/L, triglyceride levels surpassing 1.69 mmol/L, and cholesterol levels greater than 5.17 mmol/L, were not included in the study. Written informed consent was obtained from all study participants prior to their inclusion in the study. Institutional review board (IRB) approval was obtained from Jordan University Science and Technology’s review board (IRB number: 48/2022). This study was supported by an internal grant from Jordan University of Science and Technology. The authors declare that there are no conflicts of interest related to this funding source.

### 2.2. Data Collection

Patients who agreed to participate were interviewed at the start of treatment, and data regarding age, gender, smoking status, physical activity, frequency of fast food consumption per week, family history of dyslipidemia, and comorbidities such as diabetes mellites, hypertension, and polycystic ovarian syndrome were obtained. The severity of acne, its distribution, and the number of inflammatory lesions were recorded by the inspecting dermatologist. Details regarding isotretinoin treatment, such as the duration of acne vulgaris, past isotretinoin treatment, and the number of sessions taken were also obtained.

Baseline lipid profile, liver enzymes, and complete blood count were reviewed, and those with abnormalities at baseline were excluded. For those included in the study, isotretinoin was delivered via a dose from 0.25 to 1 mg/Kg of body weight. We obtained regular follow-up labs during the duration of therapy. Clinical and laboratory monitoring were performed on study participants one month after they began taking isotretinoin and one month later if their daily dosage was increased. Once the maximum dosage had been reached, patients were monitored every two months until a cumulative dose of 120–150 mg/kg was reached. However, monthly labs were performed on individuals who developed dyslipidemia until they reached the same target cumulative dose.

### 2.3. Study End Points

In this study, our primary objective is to investigate the incidence of dyslipidemia among individuals undergoing treatment with isotretinoin for acne vulgaris. Dyslipidemia was defined as having HDL levels in males of < 1.04 mmol/L, females < 1.29 mmol/L, LDL levels > 4.12 mmol/L, triglyceride > 2.26, and cholesterol > 6.19 mmol/L. Additionally, we aim to discern the risk factors that may contribute to the development of dyslipidemia in this patient population. Moreover, our secondary objective is to examine the influence of dose reduction on dyslipidemia in patients receiving isotretinoin.

### 2.4. Statistical Analysis

Statistical analysis was performed using SPSS 28.0. Simple frequencies were obtained. Continuous variables were outlined as mean ± standard deviation (SD). Categorical values were reported as percentages. We compared those that developed dyslipidemia and those that did not using Chi-square tests for categorical variables and independent t-tests for continuous variables. The logistic regression model was used to assess risk factors for the development of dyslipidemia. We further analyzed those that developed dyslipidemia using paired sample t-tests to evaluate the lipid panel before and after dose reduction. A *p*-value < 0.05 was considered significant.

## 3. Results

### 3.1. Participants

A total of 498 patients with severe acne vulgaris being initiated on isotretinoin enrolled in our study. The mean age of the participants was 20.74 (±4.07) years. Most of the participants were female (76.3%), and the average weight and height of the participants were 63.14 kg and 1.65 m, respectively. The average BMI of the participants was 23.22 kg/m^2^, with a standard deviation of 3.95 (Table 1).

About 72.5% of the participants reported a family history of acne, and 17.1% reported a family history of dyslipidemia. Only 4% of the participants had a history of polycystic ovarian syndrome. About 17.3% of the participants reported tobacco use. Regarding the location and severity of acne among the study participants, just over half the patients had acne on sites other than just the face. The severity of acne was classified as grade A (>10 nodules) in 83.3% of participants and grade B (<10 nodules) in 16.7% of participants. The mean age of onset of acne was 16.84 (±3.86) years.

Regarding lifestyle dietary and exercise habits in our study, participants reported the number of days per month that they engaged in exercise (0 = no exercise at all, 1 = 1–2 times per week, and 2 = 3 or more times per week), the number of days per month that they consumed dessert (17.51 days), the frequency of high protein consumption (10 participants reported consuming high protein), the number of days per month that they consumed dairy (22.3 days), and the number of days that they consumed fast food (5.65 days).

### 3.2. Rate and Risk Factors for Dyslipidemia

A total of 57 (11.4%) patients on isotretinoin developed dyslipidemia (Table 2). The onset of dyslipidemia was at 3.23 (±2.13) months. Regarding lipid parameters, the mean HDL level was 1.0370 mmol/L (±0.19386) and the majority of participants (75.4%) had low HDL levels, falling below the defined thresholds for both females and males. The mean LDL level was 3.014 mmol/L (±0.962), with 15.8% of participants having borderline high levels and 12.3% having high levels. Triglyceride levels showed a mean of 2.550 mmol/L (±0.461), with 21.1% of participants having borderline high levels and 78.9% having high levels. None of the participants exhibited very high triglyceride levels. The mean cholesterol level was 5.191 mmol/L (±0.908) and 17.5% of participants had borderline high levels, while another 17.5% had high levels (Table 3).

In the univariate analysis, the following variables showed a significant association with the risk of dyslipidemia: Body Mass Index (BMI) (OR 1.07, 95% CI [1.01, 1.14], *p* = 0.025) and being a smoker (OR 2.29, 95% CI [1.23, 4.27], *p* = 0.009). Gender approached statistical significance (*p* = 0.072), with females having a lower risk of dyslipidemia compared to males. Other factors, such as age, family history of dyslipidemia, family history of acne, exercise, acne onset, acne site, fast food consumption, dairy use, high protein intake, high sweet intake, and acne severity, did not show a significant association with dyslipidemia (Table 4).

In the multivariate analysis, after adjusting for potential confounding variables, being a smoker remained significantly associated with an increased risk of dyslipidemia (OR 1.97, 95% CI [1.01, 3.82], *p* = 0.046). BMI showed a trend towards significance (*p* = 0.060), suggesting that a higher BMI may contribute to an increased risk of dyslipidemia. Gender, exercise, and high sweet intake also showed trends towards significance, but did not reach statistical significance in the multivariate model (Table 4).

### 3.3. Dose Reduction Impact on Lipid Panel

**Figure 1:**
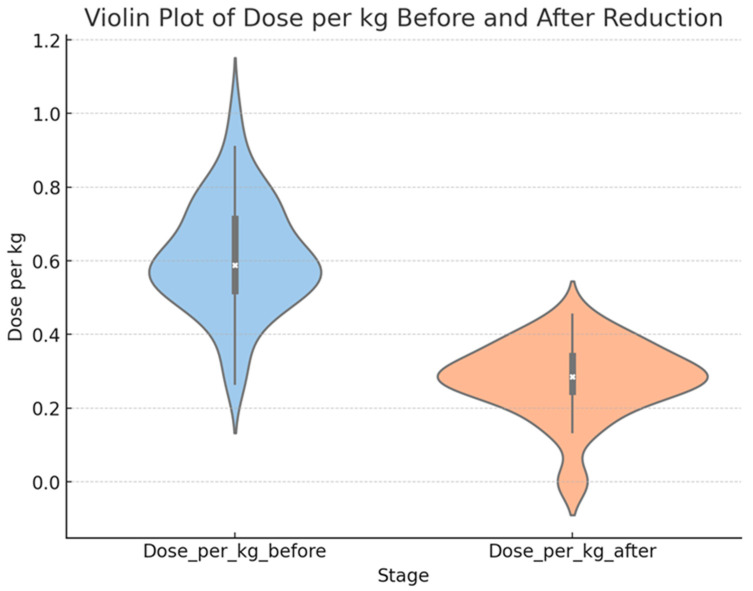
Dose of isotretinoin was reduced by approximately 50%.

Among the different dosage options, the majority of patients (86%) received a dose of 0.5–075 mg/kg. A total of 52 patients out of the 57 had a dose reduction of 50%, as shown in (Figure 1). This dose reduction was found to significantly decrease triglyceride levels primarily. However, there were no significant changes observed in the HDL (*p* = 0.392), LDL (*p* = 0.321), or cholesterol (*p* = 0.081) levels following the dose reduction (Table 5).

## 4. Discussion

This prospective observational study aims to assess the incidence of dyslipidemia and identify its risk factors in patients undergoing isotretinoin treatment. Out of the 498 patients included in the study, 57 (11.4%) developed dyslipidemia. We found a significant association between dyslipidemia and smoking, as well as higher BMI. Interestingly, reducing the dosage of isotretinoin by almost half resulted in a significant decrease in triglyceride levels. To the best of our knowledge, no previous studies have investigated the risk factors for dyslipidemia associated with isotretinoin use in the management of severe acne vulgaris.

Isotretinoin, an FDA-approved medication for severe nodulocystic acne, has been widely recognized as the most effective therapy for acne over the past four decades [1,8,9]. However, isotretinoin use is associated with various potential adverse effects, including cheilitis, benign intracranial hypertension, thrombocytopenia, skeletal hyperostosis, depression, and ocular side effects [10]. Isotretinoin has been associated with lipid elevation, although the exact underlying mechanism remains unclear [2]. Two hypotheses propose that retinoids bind to plasma albumin, displacing triglycerides, or that isotretinoin interacts with key proteins/enzymes involved in lipid metabolism [11].

In our study, 11.4% of patients on isotretinoin developed dyslipidemia, despite not having any lipid abnormalities at baseline. It is important to note that rates of isotretinoin-induced dyslipidemia vary widely in the literature, which can be attributed to differences in population specific characteristics and study designs [2,12,13,14]. Additionally, variations in dietary habits may contribute to the disparities observed in the abnormalities of serum lipid levels between our study and studies conducted in different countries [15,16,17]. Understanding these factors is crucial for interpreting and comparing the findings across different studies.

The frequency and duration of lab monitoring for patients on isotretinoin have been a subject of debate [18,19]. In our study, we found that dyslipidemia typically developed around 3.23 months after initiating treatment. These findings align with the results reported by Oktem et al., who also observed early lipid abnormalities during isotretinoin treatment (median 1–3 months) [20]. Importantly, our findings emphasize the potential significance of the early monitoring of lipid parameters during isotretinoin therapy in promptly identifying and addressing dyslipidemia.

Regarding the characteristics of our study participants, the mean age was 20.74 years, which aligns with the existing literature indicating that acne vulgaris is more commonly observed during adolescence [21]. Moreover, most of our participants were female. While it has been reported in some studies that women may have higher rates of acne, it is essential to consider potential confounding factors, such as the greater likelihood of females seeking treatment for acne vulgaris [21,22].

Among the participants who developed dyslipidemia, a considerable proportion (78.9%) had high triglyceride levels, falling within the range of 2.26–5.64 mmol/L. This finding is consistent with previous studies that have also highlighted the impact of isotretinoin on triglyceride levels [2,14]. Moreover, a notable number of participants had borderline high cholesterol levels (17.5%), while another 17.5% had levels classified as high. This is consistent with the findings of Zane et al., which identified 30% of patients with new abnormal total cholesterol levels [14].

In terms of LDL levels, the mean was 3.01 mmol/L, with 15.8% of the participants having borderline high LDL levels, and 12.3% having levels classified as high. We observed that the mean HDL level was 1.037 mmol/L, indicating a generally low HDL level among the participants. This differs from a prior report on 60 patients on isotretinoin, where no changes were noted in the HDL levels [2]. Our study may overestimate the change in the HDL levels, as only participants with abnormal baseline cholesterol, LDL, or triglyceride levels were excluded from the study 

The observed lipid abnormalities in patients on isotretinoin may be worrisome, as elevated triglyceride, LDL, and low HDL levels are known to be associated with increased cardiovascular risks in the general population [23,24]. Although the exact impact of isotretinoin-induced lipid panel abnormalities on cardiovascular risk is not fully established, some reports suggest that significant elevations in triglyceride levels during isotretinoin therapy may contribute to a higher risk of developing hyperlipidemia and metabolic syndrome in the future [3,25].

Dyslipidemia in the general population can be influenced by various factors, including age, BMI, alcohol consumption, and lifestyle choices [26,27]. In our study, the average BMI of the participants was 23.22, indicating a predominantly healthy weight status. However, it is worth noting that BMI demonstrated a trend towards significance in the multivariate analysis, suggesting a potential association between higher BMI and increased risk of dyslipidemia. Age, on the other hand, did not show a significant impact on the lipid profile. Given that our population primarily consisted of younger individuals, it was challenging to evaluate the specific influence of age on dyslipidemia in this context.

Our study also explored various lifestyle habits, including exercise and dietary factors, such as dessert consumption, high protein intake, dairy consumption, and fast food consumption. While these factors did not show a significant association with dyslipidemia, it is worth noting that exercise and high sweet intake showed trends toward significance in the multivariate analysis. Further research with larger sample sizes may help elucidate the role of these lifestyle factors in dyslipidemia development during isotretinoin therapy. Interestingly, we found that 17.3% of the participants reported tobacco use, which is a known risk factor for various health conditions, including dyslipidemia [26,28,29,30]. Smoking remained significantly associated with an increased risk of dyslipidemia even after adjusting for potential confounding variables in the multivariate analysis. If future research supports our findings, it may be reasonable to consider more frequent lipid level monitoring for individuals who smoke and incorporate smoking cessation interventions as part of the comprehensive management of acne patients undergoing isotretinoin therapy.

The incidence of blood lipid abnormalities is higher when isotretinoin is used at normal or higher doses [13]. Moderate to severe abnormalities in lipid and transaminase levels were usually temporary and reversible [14]. For the majority of those that developed dyslipidemia, a dose reduction strategy was followed. A dose reduction of isotretinoin by half was associated with a reduction in triglyceride levels, which may indicate that we are potentially able to pursue dose reduction for patients who develop dyslipidemia instead of a discontinuation of therapy in a subset of patients who may have failed other therapy options. In addition, it may be a better measure before considering lipid-lowering agents. However, we did not observe significant changes in the HDL, LDL, or cholesterol levels following the dose reduction.

### 4.1. Implications of Findings

The results of this study provide valuable insights into the incidence and risk factors of dyslipidemia in patients undergoing isotretinoin therapy. This understanding enables healthcare providers to identify individuals at higher risk and implement appropriate measures for monitoring and managing dyslipidemia during isotretinoin treatment. Moreover, this study offers crucial information for the management of dyslipidemia in individuals receiving this medication. The findings may have significant implications for the clinical management of acne vulgaris and the prevention of possible adverse cardiovascular outcomes in this population.

### 4.2. Limitations

Our study, while providing valuable insights, has several limitations that warrant consideration. Firstly, the single-center nature of the study may limit the generalizability of our findings to broader settings and populations. Additionally, the lack of diversity within our patient cohort, encompassing variations in BMI, age groups, racial backgrounds, and other baseline characteristics, may restrict the broader applicability of our results. Furthermore, the relatively small number of patients who developed dyslipidemia may limit the statistical power of our analysis when assessing risk factors and predictors. The absence of a control group further complicates the assessment of the impact of the medication. Lastly, it is worth noting that although we assumed that all triglyceride and total cholesterol samples were obtained from fasting patients, some measurements may have been influenced by inadequate adherence to fasting instructions, introducing an element of uncertainty.

## 5. Conclusions

More than 1 out of 10 of our patients on isotretinoin for the management of severe acne vulgaris developed dyslipidemia. Tobacco use was significantly associated with its development. Dose reduction effectively lowered triglyceride levels. These findings highlight the need for monitoring lipid parameters and implementing strategies like smoking cessation interventions and dose adjustments to mitigate dyslipidemia risk during isotretinoin therapy for severe acne vulgaris.

## Figures and Tables

**Table 1:** Patient characteristics of study cohort.

Variable	All
Age	20.74 (±4.07)
Female	380 (76.3%)
Male	118 (23.7%)
Weight	63.14 (±12.47)
Height	1.65 (±0.095)
Body Mass Index	23.22 (±3.95)
Family history of acne	361 (72.5%)
Family history of dyslipidemia	85 (17.1%)
History of PCOS	20 (4%)
Tobacco use	86(17.3%)
Acne site	
Only face	245 (49.2%)
Back and face	187 (37.6%)
Chest and face	20 (4%)
Shoulder and face	9 (1.8%)
Face and neck	1 (0.2%)
More than >2	35 (7%)
Other	1 (0.2%)
Severity	
A (<10 nodules)	415 (83.3%)
B (>10 nodules)	83 (16.7%)
Age of onset of acne	16.84 (±3.86)
Exercise	
No exercise	305
1–2 times/week	125
3 or more times/week	68
Dessert consumption, number of days per month	17.51 (±14.59)
High protein	10 (2%)
Dairy consumption, number of days per month	22.22 (±14.87)
Fast food consumption, number of days per month	5.72 (±6.37)

**Table 2:** Difference in baseline characteristics between those that developed dyslipidemia and those that did not.

Variable	No Dyslipidemia N = 441 (88.6%)	Dyslipidemia N = 57 (11.4%)	*p*-Value
Age	20.76 (±4.01)	20.65 (±4.52)	0.853
Female	342 (77.6%)	38 (66.7%)	0.069
Male	99 (22.4%)	19 (33.3%)	
Weight	62.63 (±12.24)	67.09 (±13.59)	0.011
Height	1.65 (±0.10)	1.66 (±0.09)	0.483
Body Mass Index	23.07 (±3.95)	24.35 (±3.82)	0.022
Family history of acne	319 (72.3%)	42 (73.7%)	0.830
Family history of dyslipidemia	73 (16.6%)	12 (21.1%)	0.396
History of PCOS	16 (3.6%)	4 (7%)	0.417
Tobacco use	69 (15.6%)	17 (29.8%)	0.008
Acne site			0.784
Only face	219 (49.7%)	26 (45.6%)	
Back and face	163 (37%)	24 (42.1%)	
Chest and face	17 (3.9%)	3 (5.3%)	
Shoulder and face	7 (1.6%)	2 (3.5%)	
Face and neck	1 (0.2%)	0 (0%)	
More than >2	33 (7.5%)	2 (3.5%)	
Other	1 (0.2%)	0 (0%)	
Severity			0.571
A (<10 nodules)	366 (83%)	49 (86%)	
B (>10 nodules)	75 (17%)	8 (14%)	
Age of onset of acne	16.83 (±3.741)	16.88 (±4.744)	0.931
Exercise			0.122
No exercise	263 (59.6%)	42 (73.7%)	
1–2 times/week	115 (26.1%)	10 (17.5%)	
3 or more times/week	63 (14.3%)	5 (8.8%)	
Dessert consumption, number of days per month	17.85 (±14.693	14.95 (±13.58)	0.158
High protein	9 (2%)	1 (1.8%)	0.885
Dairy consumption, number of days per month	22.35 (±15.26)	21.25 (±11.52)	0.599
Fast food consumption, number of days per month	5.65 (±6.12)	6.23 (±8.08)	0.520

**Table 3:** Characteristics of those that developed dyslipidemia.

	Frequency (Percent) or Mean (SD)
Relapse	11 (19.3%)
Previous isotretinoin use	5 (8.8%)
Age of prior isotretinoin use, year	19.4 (±3.51)
Onset of HLD, months	3.2281 (±2.13)
Dose:	
0.25–0.49 mg/kg	9 (15.7%)
0.5–0.75 mg/kg	39 (68.4%)
0.76–1 mg/kg	9 (15.7%)
Mean HDL, mmol/L	1.04 (±0.19)
Low (males < 1.04 mmol/L, females < 1.29 mmol/L)	43 (75.4%)
Mean LDL, mmol/L	3.01 (±0.96)
Borderline high (3.37–4.12 mmol/L)	9 (15.8%)
High (>4.12 mmol/L)	7 (12.3%)
Mean triglyceride, mmol/L	2.55 (±0.46)
Borderline high (1.69–2.25 mmol/L)	12 (21.1%)
High (2.26–5.64 mmol/L)	45 (78.9%)
Very High (>5.64 mmol/L)	0
Mean cholesterol, mmol/L	5.19 (±0.91)
Borderline high (5.17–6.19 mmol/L)	10 (17.5%)
High (>6.19 mmol/L)	10 (17.5%)

HDL: high-density lipoprotein; LDL: low-density lipoprotein.

**Table 4:** Regression model examining predictors of dyslipidemia.

	Univariate		Multivariate	
Comorbidities	OR [95% CI]	*p*-Value	OR [95% CI]	*p*-Value
Age	0.99 [0.93, 1.07]	0.853		
Tobacco use	2.29 [1.23, 4.27]	0.009	1.97 [1.01, 3.82]	0.046
Body Mass Index	1.07 [1.01, 1.14]	0.025	1.06 [1.00, 1.13]	0.060
Male gender	1.73 [0.95, 3.13]	0.072	1.57 [0.83, 2.97]	0.167
Family history of dyslipidemia	1.35 [0.68, 2.67]	0.397		
Family history of acne	1.07 [0.57, 2.00]	0.830		
Exercise 1–2 times/week	0.55 [0.26, 1.12]	0.100	0.53 [0.25, 1.11]	0.091
Exercise 3 or more times/week	0.50 [0.19, 1.31]	0.157	0.45 [0.17, 1.21]	0.115
Higher fast food intake	1.01 [0.97, 1.06]	0.520		
Higher dairy intake	1.00 [0.98, 1.01]	0.598		
Higher protein intake	0.86 [0.11, 6.89]	0.885		
Higher sweet intake	0.99 [0.97, 1.01]	0.158	0.99 [0.96, 1.01]	0.159
Acne severity	0.80 [0.36, 1.75]	0.572		
Acne age of onset	1.00 [0.94, 1.08]	0.930		
Acne site	0.95 [0.76, 1.18]	0.655		

**Table 5:** Dose reduction effect on dyslipidemia.

	Prior to Reduction (n = 52)	After Reduction (n = 52)	Paired Sample *t*-Test *p*-Value
HDL, mmol/L	1.04 (±0.20)	1.066 (±0.24)	0.392
LDL, mmol/L	2.97 (±0.96)	3.085 (±0.97)	0.321
Cholesterol, mmol/L	5.14 (±0.91)	4.931 (±0.97)	0.081
Triglyceride, mmol/L	2.53 (±0.40)	1.686 (±0.53)	<0.001

HDL: high-density lipoprotein; LDL: low-density lipoprotein.

## Data Availability

The datasets presented in this article are not readily available because they are part of an ongoing study and contain sensitive patient information. Requests to access the datasets should be directed to Jihan Muhaidat at jmmuhaidat@just.edu.jo.

## Abbreviations

BMI:	Body Mass Index
CI:	Confidence Interval
DLP:	Dyslipidemia
HDL:	High-density lipoprotein
HLD:	Hyperlipidemia
IRB:	Institutional review board
LDL:	Low-density lipoprotein
PCOS:	Polycystic ovarian syndrome
SD:	Standard deviation
SPSS:	Statistical Package for the Social Sciences
TG:	Triglycerides
